# Investigating the effects of Pleistocene events on genetic divergence within *Richardsonius balteatus*, a widely distributed western North American minnow

**DOI:** 10.1186/1471-2148-14-111

**Published:** 2014-05-23

**Authors:** Derek D Houston, Dennis K Shiozawa, Brian Tilston Smith, Brett R Riddle

**Affiliations:** 1 School of Life Sciences, University of Nevada-Las Vegas, Las Vegas, NV 89154-4004, USA; 2 Department of Biology, Brigham Young University, Provo, UT 84602, USA; 3 Museum of Natural Science, Louisiana State University, Baton Rouge, LA 70803, USA; 4 Current address: Department of Ecology, Evolution, & Organismal Biology, Iowa State University, Ames, IA 50011, USA; 5 Current address: Department of Ornithology, American Museum of Natural History, Central Park West at 79th Street, New York, NY 10024, USA

**Keywords:** Phylogeography, Redside shiner, Pluvial lakes, Glacial cycles, Climate change, Post-glacial colonization

## Abstract

**Background:**

Biogeographers seek to understand the influences of global climate shifts and geologic changes to the landscape on the ecology and evolution of organisms. Across both longer and shorter timeframes, the western North American landscape has experienced dynamic transformations related to various geologic processes and climatic oscillations, including events as recently as the Last Glacial Maximum (LGM; ~20 Ka) that have impacted the evolution of the North American biota. Redside shiner is a cyprinid species that is widely distributed throughout western North America. The species’ native range includes several well-documented Pleistocene refugia. Here we use mitochondrial DNA sequence data to assess phylogeography, and to test two biogeographic hypotheses regarding post-glacial colonization by redside shiner: 1) Redside shiner entered the Bonneville Basin at the time of the Bonneville Flood (Late Pleistocene; 14.5 Ka), and 2) redside shiner colonized British Columbia post-glacially from a single refugium in the Upper Columbia River drainage.

**Results:**

Genetic diversification in redside shiner began in the mid to late Pleistocene, but was not associated with LGM. Different clades of redside shiner were distributed in multiple glacial age refugia, and each clade retains a signature of population expansion, with clades having secondary contact in some areas.

**Conclusions:**

Divergence times between redside shiner populations in the Bonneville Basin and the Upper Snake/Columbia River drainage precedes the Bonneville Flood, thus it is unlikely that redside shiner invaded the Bonneville Basin during this flooding event. All but one British Columbia population of redside shiner are associated with the Upper Columbia River drainage with the lone exception being a population near the coast, suggesting that the province as a whole was colonized from multiple refugia, but the inland British Columbia redside shiner populations are affiliated with a refugium in the Upper Columbia River drainage.

## Background

A major goal of biogeography is to investigate the influences of geological and climatic changes on the divergence and distribution of populations, species, and higher taxa. A reasonable expectation in biogeographic studies is that episodes of geographic isolation, and consequently, opportunities for divergence should be favored on topographically complex landscapes [1,2]. The complex western North American landscape has a dynamic history of dramatic alterations resulting from tectonic processes as well as Pleistocene glacial cycles. These geological and climatic processes have influenced patterns of gene flow and diversification across a wide variety of taxa [3-9]. Such processes have heavily influenced the evolution of the North American fish fauna as well [10-15]. In western North America, the evolution of freshwater fishes has resulted from long term isolation of populations [16], but with intermittent dispersal occurring between hydrological basins for at least some taxa during major events such as stream captures and floods, some of which have been facilitated by climatic events [10,17-21].

**Figure 1:**
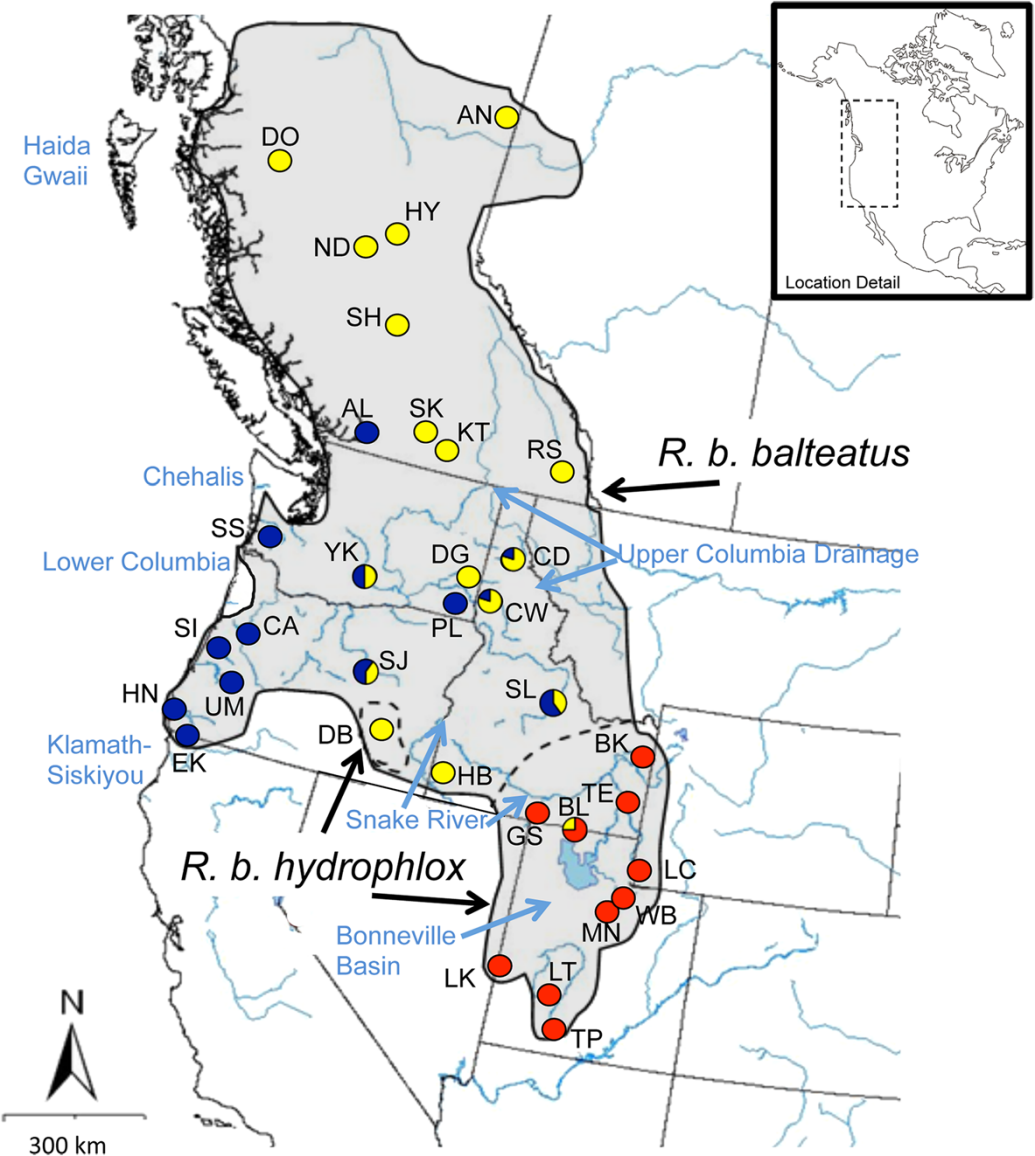
**Distribution map for *R. balteatus*.** The dashed lines represent subspecies distribution boundaries. Circles represent sampling localities, and are color coded according to clade: Bonneville/Upper Snake (red), Northern Inland (yellow), and Pacific Northwestern lineages (blue). Population abbreviations correspond to those listed in Table 1. Six populations show a mixture of haplotypes, and the proportions are represented as pie charts. Documented Pleistocene refugia and major rivers are also labeled.

Climatic oscillations during the Pleistocene have played a significant role in the evolution of the western North American biota by forcing organisms through a series of range contractions and expansions, range shifts, or localized extinctions as glacial ice sheets expanded and retracted [7]. In northwestern North America, refugia are postulated to have occurred in several areas, including Beringia [22-25], the Chehalis River Valley [4,26-28], the lower Columbia River drainage [29-31], the Upper Columbia River drainage [32-36], Haida Gwaii (a.k.a., Queen Charlotte Islands: an archipelago off the Pacific Coast of British Columbia) [37-39], and the Klamath-Siskiyou region [5,36,40-42] (Figure 1). Additionally, some taxa have retained genetic signatures of survival in northern and southern refugia along the Pacific Coast [27,43,44].

In addition to ice sheets, glacial-age lakes associated with the ice sheets altered the landscape of western North American in ways that may have affected gene flow. Perhaps the best studied of these is Lake Missoula, a glacial lake that formed in western Montana when the Clark Fork River was dammed by a lobe of the Cordilleran Ice Sheet [45]. Catastrophic flooding occurred when the ice dam broke, and the rushing waters carved the channeled scablands of eastern Washington [18,45-47]. This process is hypothesized to have occurred approximately 40 (and perhaps as many as 100) times during the Pleistocene [48]. While most of those flooding events were small, some were catastrophic [48]. The highest magnitude Missoula floods were responsible for the capture of the Palouse River (from the Columbia River) by the lower Snake River, and the formation of Palouse Falls which now stand as a barrier to dispersal for aquatic taxa [49], likely affecting patterns of gene flow for many of the aquatic taxa in the area. Glacial floods were also responsible for completely filling the Willamette Valley in western Oregon [18], an event that appears to have influenced genetic structure for at least some taxa by forming a barrier between populations of terrestrial organisms [50], and possibly providing more connectivity of aquatic habitats and thus more opportunities for aquatic organisms to disperse. Glacial lakes and similar flooding events were also operating in northern areas (i.e., British Columbia) as the ice sheets retreated at the end of the Pleistocene [18].

The increased precipitation and decreased evaporation associated with glacial maxima resulted in the formation of pluvial lakes in many of the valleys of the Basin and Range province during the Pleistocene. Pluvial Lake Bonneville in the Bonneville Basin experienced lake high-stands coincident with glacial melt waters [51]. The highest level reached by Lake Bonneville is attributed to the capture of the Bear River from the Upper Snake River into the Bonneville Basin approximately 35,000 years ago [10,19,52]. The influx of water from the Bear River caused Lake Bonneville to rise until approximately 14,500 years ago when the lake breached a northern sill in Red Rocks Pass Idaho. Lake Bonneville drained catastrophically into the Upper Snake River in an event known as the Bonneville Flood [51,53]. This and similar pluvial lake outflows have been hypothesized to be important inter-basin transfer mechanisms for fish and other aquatic taxa [19,54,55].

One western North American fish species, redside shiner *Richardsonius balteatus* (Richardson), occurs in many areas heavily influenced by Pleistocene climatic oscillations, either by glaciation directly, or by glacial and pluvial lakes. The species’ native range spans the Bonneville Basin and the Snake-Columbia River basins west of the Rocky Mountains, ranging from southern Utah to northern British Columbia, and westward to the Pacific Coast of Oregon, Washington and British Columbia (Figure 1). Phylogenetic analyses based on mitochondrial DNA (mtDNA) data have demonstrated that *R. balteatus* is a monophyletic species that exhibits phylogeographic structure [55]. Pronounced morphological variation occurs among natural populations [10,18,19]. Variation in the number of anal fin rays, along with geographic distribution are the primary considerations for dividing the species into two subspecies, *R. b. balteatus* and *R. b. hydrophlox*. Such morphological variation could be the result of genetic differences resulting from historical isolation among populations, or of phenotypic plasticity in the face of different selective pressures in different environments. Moreover, subspecies *R. b. hydrophlox* exhibits geographic variation in somatic growth rates, at least some of which reflects genetic differences between populations [56].

*Richardsonius balteatus* is estimated to have diverged from its sister species, Lahontan redside shiner *Richardsonius egregius* (Girard)*,* during the Pliocene [55,57], but genetic divergence within *R. balteatus* occurred more recently, during the early to mid Pleistocene and the species exhibits relatively shallow phylogenetic structure [55]. Such shallow structure is often attributed to influences of late Quaternary climatic shifts on patterns of gene flow. Because many parts of the current range of *R. balteatus* were affected by Pleistocene climatic oscillations, well-documented changes in hydrological connections are postulated to have influenced range expansion. The southernmost portion of the range, where *R. b. hydrophlox* occurs, includes the Bonneville Basin and Upper Snake River Plain (the subspecies also has disjunct populations in southeast Oregon; Figure 1).

Hubbs and Miller [54] postulated that *R. balteatus* invaded the Bonneville Basin from the Snake River at the time of the Bonneville Flood because it occurred above barrier falls in northern parts of its range. This proposed dispersal pathway is opposite to the direction of this massive flooding event, but Hubbs and Miller stated that fish moved in both directions through this connection. Their scenario of a southward invasion of the Bonneville Basin at the time of the Bonneville Flood is consistent with the fossil record, which shows that the extinct taxon *R. durranti* was present in Pliocene sediments of Lake Idaho [58], indicating that the genus occurred in the Snake River system in the Pliocene. The earliest known *R. balteatus* fossil in the Bonneville Basin is late Pleistocene in age [59]. However, fossil data merely place a minimum age for the occurrence of taxa in an area, so it is possible that *R. balteatus* entered the Bonneville Basin from the Snake River earlier in the Pleistocene, or has been in the Bonneville Basin for a much longer period of time. Molecular data have shown that both the Bear River capture and the Bonneville Flood transferred some freshwater fishes [17,52], but the biogeography of other cyprinids suggests older (Miocene) connections between the Upper Snake River and Bonneville Basin existed [11,20,60,61].

*Richardsonius balteatus* populations in previously glaciated areas must have colonized post-glacially. It is hypothesized that *R. balteatus* colonized the northernmost parts of its range (i.e., British Columbia) post-glacially from a refugium in the Upper Columbia River Basin based on current distributions above barrier falls and several known geomorphic connections [18,19]. The Upper Columbia River drainage (including the Clearwater and Salmon rivers) is a hypothesized refugial area for fishes, amphibians, and plants [18,27,32,33,35], and could have been for *R. balteatus* as well. However, the current distribution of *R. balteatus* includes many proposed refugia other than the Upper Columbia River drainage, including the Chehalis River Valley, the lower Columbia River, and areas along the Oregon Coast. It is unknown whether *R. balteatus* colonized the aforementioned refugial areas post-glacially, or if the species occurred in one or more of them throughout the Pleistocene. Some phylogeographic evidence supports the latter scenario based on divergence times among three clades that date to the early to mid Pleistocene [55]. If the species did occur in Pleistocene refugia other than the Upper Columbia River drainage during the Pleistocene, then the species may have colonized northern environments from outside the Upper Columbia River system.

Herein, we use mtDNA sequence data to test two hypotheses regarding proposed range expansion events by *R. balteatus*: 1) We test the hypothesis that the species invaded the Bonneville Basin at the time of the Bonneville Flood [54] by assessing whether a pattern consistent with such a scenario exists. If *R. balteatus* did enter the Bonneville Basin at that time, then Bonneville Basin populations should exhibit signs of recent rapid expansion, and molecular dating estimates should be consistent with a late Pleistocene divergence time between Bonneville Basin populations and those in the Upper Snake/Columbia River drainage. 2) We test the hypothesis that *R. balteatus* colonized British Columbia post-glacially from a single refugium in the Upper Columbia River drainage [18]. If *R. balteatus* expanded northward into British Columbia from a single refugium, then genetic diversity in northernmost populations would be expected to be low, and should resemble that of the ancestral population. If *R. balteatus* expanded from multiple refugia, then haplotypes observed in previously glaciated areas should resemble those of each of the refugia from which *R. balteatus* dispersed. Furthermore, if *R. balteatus* expanded from multiple refugia, then greater genetic diversity would be expected in some populations representing suture zones where genetic admixture may have occurred. Lastly, we discuss an overview of the phylogeography of the species.

To test these hypotheses, we generated a phylogeny using maximum likelihood and Bayesian inference to assess the evolutionary relationships among redside shiner populations. We also generated a haplotype network to visualize the finer-scale phylogeographic structure. We estimated divergence times and evaluated changing population sizes through time to determine if divergence times and population expansions were consistent with late Pleistocene events.

## Methods

### Sampling

We compiled a dataset comprising 157 individuals from thirty-four populations throughout the native range of *R. balteatus*, along with 23 *R. egregius* individuals from five populations that were used as the outgroup (Table 1). We used previously published DNA sequences from 28 of those populations [55], and generated DNA sequence data for 30 individuals from 6 additional populations. We sampled individuals from those six populations using a backpack electroshocker, a beach seine, or baited minnow traps. Once we sampled fish, we euthanized them by administering a lethal overdose of tricaine methanesulfonate (MS-222), then immediately placed whole specimens into 95% ethanol to preserve tissues for genetic analyses. We placed the ethanol preserved samples on ice and transported them to the University of Nevada, Las Vegas (UNLV) where we assigned Monte L. Bean Life Science Museum (MLBM) catalogue numbers and Las Vegas Tissue (LVT) numbers to them before taking tissues for genetic analysis. All specimens were deposited in the MLBM fish collection as vouchers at the completion of this study, and all specimens also have a sample of muscle tissue stored in the LVT collection at UNLV. The UNLV Animal Care and Use Committee approved the protocols for the whole project, including the sampling and sacrificing of these minnows for the purpose of this research, as well as all subsequent handling of tissues/DNA samples (IACUC Protocol #R701-0703-179). We performed all collections under the appropriate state and provincial permits (issued to DDH and DKS).

**Table 1:** Sampling localities

**Sampling locality**	**Latitude/longitude**	**LVT #**	**MLBM #**	**GenBank #**	**N**
**BRITISH COLUMBIA**					
Alouette Lake (AL)*,	49.290 N, 122.488 W	9751 – 9755	63987 – 63991	cyt *b*: KJ468400 – KJ468404	5
Fraser River Drainage				CR: KJ468430 – KJ468434	
Lower Mainland Region					
Antonelli Creek (AN),	56.334 N, 120.154 W	9721 – 9725	63951 – 63955	cyt *b*: GU182709 – GU182713	5
Peace River Drainage				CR: GU182504 – GU182508	
Peace Region					
Doris Lake (DO),	54.945 N, 126.552 W	9731 – 9735	63965 – 63969	cyt *b*: GU182743 – GU182747	5
Skeena River Drainage				CR: GU182538 – GU182542	
Skeena Region					
Hay Creek (HY)*,	54.074 N, 122.368 W	9821 – 9825	112018 – 112022	cyt *b*: KJ468405 – KJ 468409	5
Fraser River Drainage				CR: KJ468435 – KJ 468439	
Omineca Region					
Kettle River (KT),	49.013 N, 118.200 W	9001 – 9005	084184 – 084188	cyt *b*: GU182777 – GU182781	5
Columbia River Drainage				CR: GU182572 – GU182576	
Okanagan Region					
Nadsilnich (West) Lake (ND)*,	53.732 N, 122.859 W	9831 – 9835	112035 – 112039	cyt *b*: KJ468410 – KJ468414	5
Fraser River Drainage				CR: KJ468440 – KJ468444	
Omineca Region					
Rosen Lake (RS)*,	49.402 N, 115.254 W	9711 – 9715	63940 – 63944	cyt *b*: KJ468415 – KJ468419	5
Columbia River Drainage				CR: KJ468445 – KJ468449	
Kootenay Region					
Shumway Lake (SH)*,	50.511 N, 120.264 W	9811 – 9815	112008 – 112012	cyt *b*: KJ468420 – KJ468424	5
Fraser River Drainage				CR: KJ468450 – KJ468454	
Thompson Region					
Similkameen River (SK),	49.175 N, 119.768 W	8991 – 8994	84171 – 84175	cyt *b*: GU182838 – GU182841	4
Columbia River Drainage				CR: GU182633 – 182636	
Okanagan Region					
**IDAHO**					
Big Bear Creek (Clearwater River; CW),	46.600 N, 116.660 W	8247 – 8251	138772 – 138776	cyt *b*: GU182738 – GU182742	5
Columbia River Drainage				CR: GU182533 – GU182537	
Latah County					
Blackfoot River (BK),	43.230 N, 112.030 W	7851 – 7855	58911 – 58915	cyt *b*: GU182714 – GU182718	5
Upper Snake River Drainage				CR: 182509 – GU182513	
Bingham County					
Cold Creek (Goose Creek; GS),	42.093 N, 113.933 W	7314 – 7318	61222 – 61226	cyt *b*: GU182757 – GU182761	5
Upper Snake River Drainage				CR: GU182552 – GU182556	
Cassia County					
Coeur d’Alene River (CD),	47.553 N, 116.257 W	6301 – 6305	63627 – 63631	cyt *b*: GU182733 – GU182737	5
Columbia River Drainage				CR: GU182528 – GU182532	
Shoshone County					
Hurry Back Creek (HB),	42.581 N, 116.676 W	7861 – 7865	63743 – 63747	cyt *b*: GU182762 – GU182766	5
Lower Snake River Drainage				CR: GU182557 – GU182561	
Owyhee County					
Salmon River (SL),	44.639 N, 114.122 W	7871 – 7875	63780 – 63784	cyt *b*: GU182833 – GU182837	5
Lower Snake River Drainage				CR: GU182628 – GU182632	
Custer County					
Teton River (TE),	43.750 N, 112.200 W	7214 – 7218	63688 – 63692	cyt *b*: GU182847 – GU182850	4
Upper Snake River Drainage				CR: GU182642 – GU182645	
Teton County					
**OREGON**					
Callapooia River (CA),	44.461 N, 123.076 W	6291 – 6295	63642 – 63646	cyt *b*: GU182728 – GU182732	5
Columbia River Drainage				CR: GU182523 – GU182527	
Linn County					
Callapooya Creek (Umpqua River; UM),	43.413 N, 123.207 W	8267 – 8271	68425 – 68429	cyt *b*: GU182860 – GU182864	5
Pacific Ocean Drainage				CR: GU182655 – GU182659	
Douglas County					
Donner und Blitzen River (DB),	42.801 N, 118.967 W	9251 – 9255	114035 – 114039	cyt *b*: GU182748 – GU182751	5
Harney Basin				CR: GU182543 – GU182546	
Harney County					
Elk Creek (EK),	42.033 N, 123.750 W	7334 – 7338	59297 – 59301	cyt *b*: GU182752 – GU182756	5
Pacific Ocean Drainage				CR: GU182457 GU182551	
Josephine County					
Hunter Creek (HN),	42.352 N, 124.353 W	7234 – 7238	63653 – 63657	cyt *b*: GU182767 – GU182771	5
Pacific Ocean Drainage				CR: GU182562 – GU182566	
Curry County					
South Fork John Day River (SJ),	44.424 N, 119.540 W	7224 – 7228	63673 – 63677	cyt *b*: GU182823 – GU182827	5
Columbia River Drainage				CR: GU182618 – GU182622	
Grant County					
Siuslaw River (SI),	44.000 N, 123.689 W	8452 – 8456	63929 – 63933	cyt *b*: GU182828 – GU182832	5
Pacific Ocean Drainage				CR: GU182623 – GU182627	
Lane County					
**UTAH**					
Beaver Creek (Weber River; WB),	40.626 N, 111.163 W	8257-58, 8260-61	69873-74, 69876-77	cyt *b*: GU182865 – GU182868	4
Bonneville Basin				CR: GU182660 – GU182663	
Summit County					
Blue Creek (BL),	41.952 N, 112.723 W	7300 – 7303	68244 – 68247	cyt *b*: GU182719 – GU182722	4
Bonneville Basin				CR: GU182514 – GU182517	
Box Elder County					
Lake Creek (LK),	38.767 N, 114.048 W	7244 – 7248	68439 – 68443	cyt *b*: GU182782 – GU182786	5
Bonneville Basin				CR: GU182577 – GU182581	
Millard County					
Little Reservoir (LT),	38.250 N, 112.480 W	7324 – 7328	63718 – 63722	cyt *b*: GU182795 – GU182799	5
Bonneville Basin				CR: GU182590 – GU182594	
Beaver County					
Main Creek (MN),	40.394 N, 111.442 W	8042 – 8046	63798 – 63802	cyt *b*: GU182810 – GU182814	5
Bonneville Basin				CR: GU182605 – GU182609	
Wasatch County					
Tropic Reservoir (TP),	37.580 N, 112.250 W	7284 – 7288	63705 – 63709	cyt *b*: GU182855 – GU182859	5
Bonneville Basin				CR: GU182650 – 182654	
Garfield County					
**WASHINGTON**					
Dragoon Creek (DG),	47.887 N, 117.433 W	7244 – 7248	63183 – 63187	cyt *b*: GU182783 – GU182786	5
Columbia River Drainage				CR: GU182577 – GU182581	
Spokane County					
North Fork Palouse River (PL),	46.920 N, 117.339 W	8551 – 8555	63622 – 63626	cyt *b*: GU182815 – GU182819	5
Lower Snake River Drainage				CR: GU182610 – GU182614	
Whitman County					
Satsop River (SS)*,	46.999 N, 123.492 W	9741 – 9745	63976 – 63980	cyt *b*: KJ468425 – KJ468429	5
Chehalis River Drainage				CR: KJ468455 – KJ468459	
Grays Harbor County					
Yakima River (YK),	46.417 N, 120.333 W	7354-6, 7358	63583-5, 63587	cyt *b*: GU182873 – GU182876	4
Columbia River Drainage				CR: GU182668 – GU182671	
Yakama County					
**WYOMING**					
LaChappelle Creek (LC),	41.127 N, 110.787 W	8078 – 8081	63810 – 63813	cyt *b*: GU182787 – GU182789	4
Bonneville Basin				CR: GU182583 – GU182584	
Uinta County					

Location information for the thirty-five populations of *R. balteatus* included in this study, along with the number of individuals from each population (N) that we included in our analyses. Population abbreviations are given in parentheses after the name of the sampling locality. Accession numbers are given for the Las Vegas Tissue collection (LVT) where muscle tissues are stored, the Monte L Bean Museum (MLBM) where whole specimens are stored, and GenBank where control region and cyt *b* sequences have been deposited.

*denote populations for which sequences were newly generated for this study.

### DNA extraction and polymerase chain reaction

We extracted whole genomic DNA from muscle tissues using the manufacturer’s recommended protocol for the Qiagen DNeasy tissue kit. We verified successful extractions qualitatively by viewing the DNA product under ultra-violet radiation following gel electrophoresis in a 0.8% agarose gel. We chose to amplify the control region (CR) and the cytochrome *b* protein coding gene (cyt *b*) of the mitochondrial genome because they are rapidly evolving markers in fishes [62], but neither of them exhibits saturation in closely related cyprinid species [63-66], and therefore they should be useful in detecting phylogeographic structure caused by Pleistocene events. Moreover, gene sequences for many populations were already available [55]. We amplified both markers via the polymerase chain reaction (PCR) using the oligonucleotide primers HA-a and LA-a for cyt *b*[67], and L-PRO and MRT-2 for CR [68,69]. We mixed reaction cocktails for PCR using approximately 100.0 ng DNA template, 10.0 pmoles of each oligonucleotide primer, 2.25 μl of molecular grade water, and 6.25 μl of Promega GoTaq hot start green master mix for a total reaction volume of 12.5 μl. We used the following thermal profile for PCR: An initial denature of 95.0°C for four minutes, followed by thirty-five cycles of 95.0°C for 30 seconds, annealing at 50.0°C for 30 seconds, and extension at 72.0°C for 90 seconds, a final extension at 72.0°C for 7 minutes, and a rapid cool down to 4.0°C. We verified successful PCR qualitatively by viewing bands of appropriate size following electrophoresis on 0.8% agarose gels. We purified PCR products using the manufacturer’s recommended protocol for the Qiagen QiaQuick PCR purification kit.

### DNA sequencing and alignment

We used the same primers for Sanger sequencing as we used for amplifying both markers, and sequenced light and heavy strands for each. We performed cycle sequencing reactions using Big Dye chemistry. Reaction cocktails contained 3.0 μl of purified PCR product, 12.2 μl of molecular grade water, 3.2 μl of 2.5X Tris buffer, 0.8 μl of 25 mM MgCl_2_, 0.3 μl of 10.0 μM oligonucleotide primer, and 0.5 μl of dye terminator reaction mix for an overall reaction volume of 20.0 μl. The thermal profile consisted of twenty-five cycles of 96.0°C for 10 seconds, 50.0°C for 5 sec, and 60.0°C for 5 minutes followed by a 4.0° hold. In some cases it was necessary to use internal sequencing primers to complete the sequence, so for cyt *b* we used either Sq7Hrs [55] or Sq3L [61], and for CR we used 12Rrs and CR7H [55]. We removed excess dye terminators from cycle sequencing products using G-50 Fine Sephadex™ in Centri-Sep™ spin columns, and performed all sequencing on an ABI 3130 automated sequencer.

We aligned sequences using the automatic assembly function in Sequencher v. 4.8 (Gene Codes Corp.) then inspected the aligned sequences by eye and made corrections manually. We used amino acid sequence and a *R. balteatus* cyt *b* sequence [GenBank: AY096011] as references for aligning and editing cyt *b* sequences. There were no gaps in the final cyt *b* alignment, but there were in the final CR alignment. Individuals carried between three and five insertion/deletion sequences (each one bp long) in their non-coding CR sequences, but these were straightforward to align. Because both CR and cyt *b* are in the mitochondrial genome, and are thus inherited as a unit, we concatenated the sequences prior to performing phylogenetic analyses.

### Phylogenetic analyses

To get a broad assessment of redside shiner phylogeography, we generated phylogenies using maximum likelihood (ML) and Bayesian inference. We selected the appropriate model of sequence evolution to be used in the phylogenetic analyses using jModeltest [70], and we reconstructed the ML phylogeny using TreeFinder (version of 2008) [71]. We performed 1000 bootstrap replicates to estimate nodal support for ML analysis. In Bayesian analysis, we employed a Markov Chain Monte Carlo approach with one cold chain and three heated chains using the program MrBayes v.3.1.2 [72]. We ran the Bayesian analysis for 10,000,000 generations, sampling every 1000 generations. We verified that the analysis reached stationarity and evaluated mixing among chains using Tracer v.1.5.0 [73]. To get appropriate levels of mixing between chains we lowered the temperature setting to T = 0.05. We discarded the first 2,500,000 generations (25%) as burn-in, and obtained posterior probabilities using a majority rule consensus of the remaining topologies.

To better visualize intraspecific genetic variation within *R. balteatus*, we created a haplotype network using the software program TCS v.1.21 [74], using the default connection limit of 95% and treating gaps as a fifth character state. In some cases it was necessary to break loops among haplotypes that were not very divergent (but never among haplotypes spanning the three major clades), which we did using three criteria that are based on coalescent theory and are outlined by Kauwe et al. [75]. These three criteria are as follows: 1) Geography – haplotypes are more likely to be closely related to those from individuals with close geographic proximity than to those from individuals that were sampled from locations that are further away. 2) Topology – haplotypes are more likely to be closely related to those branching from basal nodes in a phylogeny than they are to be closely related to haplotypes that occur in the tips of a phylogenetic tree. 3) Frequency – haplotypes are more likely to be closely related to haplotypes that are shared than they are to be closely related to those that are carried by just one individual.

### Molecular dating estimation

Molecular dating estimates allowed us to estimate whether divergence times between clades of redside shiner were consistent with the late Pleistocene time-frame of the phylogeographic hypotheses we sought to test. We performed molecular dating analyses using an uncorrelated lognormal relaxed clock in BEAST v. 1.7.5 [76] to estimate divergence times for clades within *R. balteatus*. We used the GTR + I + G model of sequence evolution (selected by jModelTest) and the coalescent constant model to set the prior on the tree. For the mutation rate prior distribution, we used a lognormal distribution with a mean rate of 1.4% sequence divergence per million years as estimated for *Richardsonius*, and specified a range of 1.0% to 2.4% sequence divergence per million years to cover the range of mutation rates for cyt *b* for closely related genera [55] as well as reported mutation rates for CR in other cyprinids [77,78]. We ran the MCMC chain for 50,000,000 generations, sampling every 1000 generations, and discarded the first 5,000,000 generations as burn-in. We verified that the program reached stationarity and that there was proper mixing of chains by viewing the results in Tracer v.1.5.0 [79]. Trees were annotated using TreeAnnotator v.1.7.2 (part of the BEAST package) [76].

### Historical demography

If redside shiner dispersed into the Bonneville Basin at the time of the Bonneville Flood, and into British Columbia from a single refugium as glaciers retreated, then redside shiner populations in these areas are predicted to exhibit signs of recent rapid expansion. We tested for recent rapid expansion in each of the three major redside shiner clades (see Results). To do so, we used BEAST v.1.7.5 [76] to generate Bayesian skyline plots that use coalescent modeling to infer population size over time [80,81]. We used a coalescent constant size tree prior, and employed a strict clock (with uniform rates across branches) using a uniform prior distribution with a mutation rate prior of 1.4% sequence divergence per million years [55], but allowing for a range of 1.0% to 2.4% sequence divergence per million years. We used the HKY substitution model of sequence evolution for each individual clade (based on the results of jModeltest). The model differed from that used in the phylogenetic analyses because model selection was run on each clade individually, and no outgroup taxa were included. We ran the analysis for 30 million generations, logging every 1000 generations, and discarded the first 3 million generations (10%) as burn-in. Each of the three lineages was analyzed separately, but the parameters were the same for all three analyses. We performed the same MCMC diagnostics as described in our divergence time analysis.

## Results

### DNA sequencing and alignment

DNA sequencing yielded 1140 bp of cyt *b* and 961 bp of CR from 157 *R. balteatus* individuals from 34 populations, and 23 *R. egregius* individuals from five populations [55,57], for a total of 180 individuals. Of those 2101 characters, 1889 were invariable, 212 were variable, and 160 of the variable characters were parsimony informative. Ninety-seven unique mtDNA haplotypes were found among all sampled populations of *R. balteatus*. All DNA sequences are available in GenBank (see Table 1 for GenBank accession numbers).

### Phylogenetic analyses

**Figure 2:**
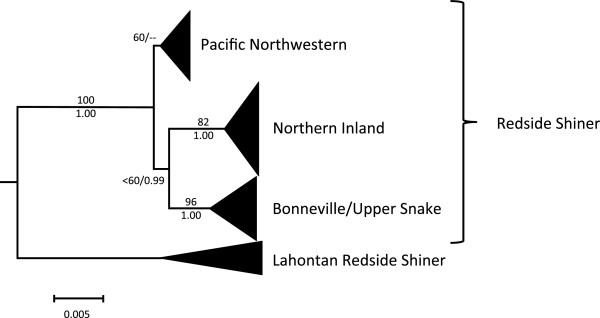
**Redside Shiner Phylogeny.** Phylogeny showing three major clades of redside shiner. Maximum likelihood bootstrap support values are listed above branches, and Bayesian posterior probabilities are listed below branches. The three clades are expanded for viewing in Figures 3, 4 and 5.

**Figure 3:**
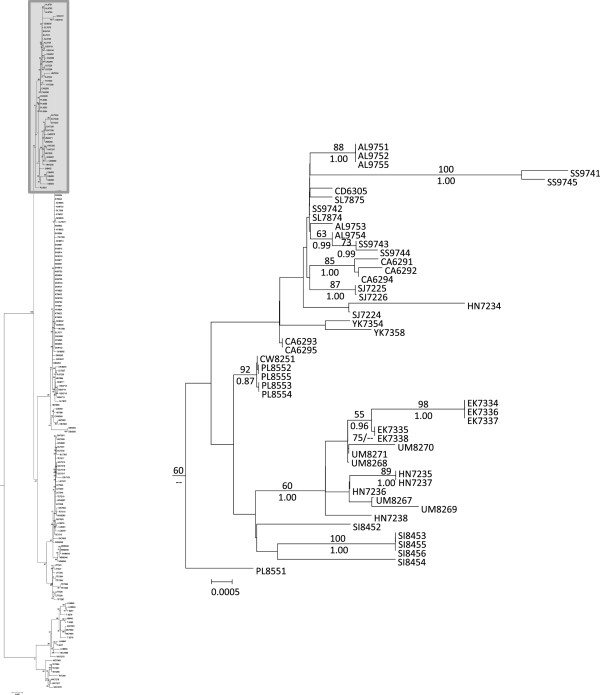
**Pacific Northwestern Lineages.** Phylogeny showing the relationships of the Pacific Northwestern lineages. Numbers above branches represent ML bootstrap values, and numbers below are posterior probabilities. Taxa are labeled with a two-letter population abbreviation (see Table 1) followed by individual LVT ID numbers. The shaded box on the left illustrates the section of the overall phylogeny that is enlarged.

**Figure 4:**
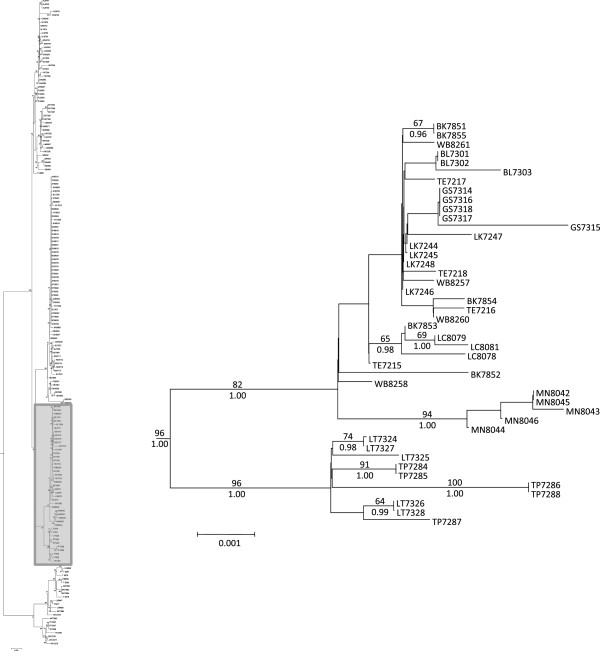
**Bonneville Basin/Upper Snake Clade.** Phylogeny showing the relationships of individuals from the Bonneville Basin/Upper Snake River drainages. Numbers above branches represent ML bootstrap values, and numbers below are posterior probabilities. Taxa are labeled with a two-letter population abbreviation (see Table 1) followed by individual LVT ID numbers. The shaded box on the left illustrates the section of the overall phylogeny that is enlarged.

**Figure 5:**
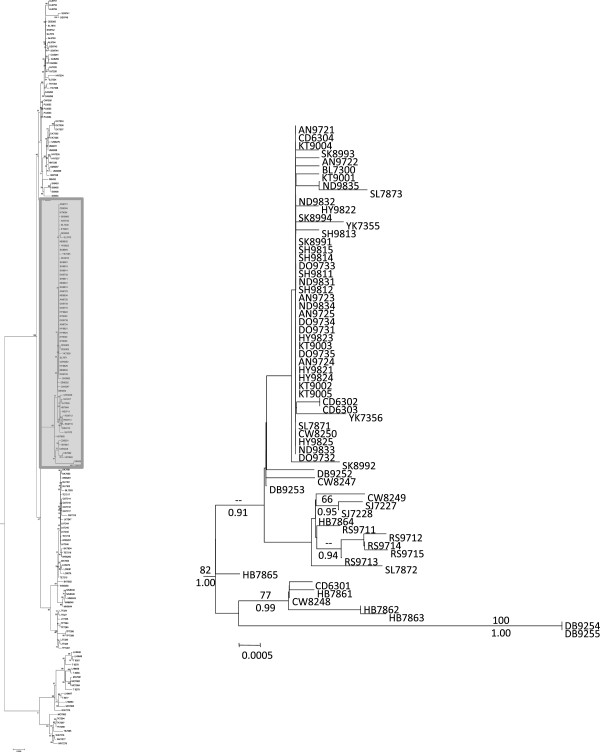
**Northern Inland Clade.** Phylogeny showing the relationships of individuals from the Upper Columbia drainages (including the lower Snake River) and inland British Columbia. Numbers above branches represent ML bootstrap values, and numbers below are posterior probabilities. Taxa are labeled with a two-letter population abbreviation (see Table 1) followed by individual LVT ID numbers. The shaded box on the left illustrates the section of the overall phylogeny that is enlarged.

The jModeltest results selected the GTR + I + G model of sequence evolution as the best fit for the concatenated mtDNA data set under the Akaike Information Criterion and the Bayesian Information Criterion. Phylogenies produced by ML and Bayesian analyses were similar, so only the ML phylogeny is shown, but ML bootstrap values and Bayesian posterior probabilities are mapped onto the nodes (Figure 2). For clarity, the major clades are presented in three different figures (Figures 3, 4 and 5).

**Figure 6:**
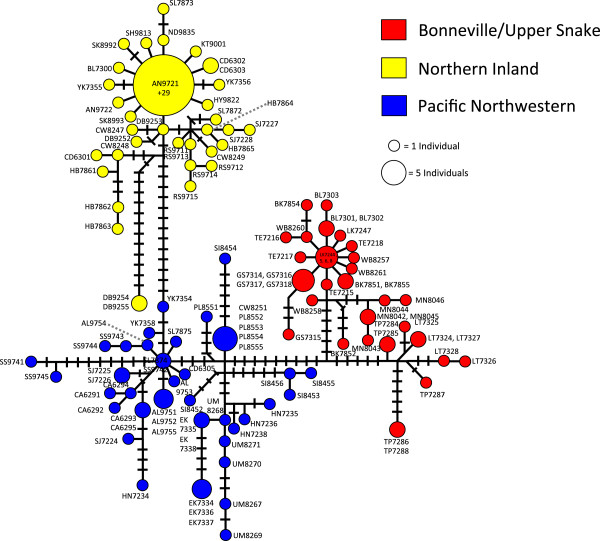
**Redside shiner mtDNA haplotype network.** Haplotype network showing the intraspecific genetic diversity for *R. balteatus*. Circles represent unique mtDNA haplotypes, and are color-coded corresponding to phylogenetic position. Circle size represents the number of individuals carrying each haplotype. Hash marks represent nucleotide base changes between haplotypes.

Pacific Northwest populations do not form a well-supported monophyletic clade although ML analysis did offer very weak support (ML bootstrap = 60) for combining these lineages into a monophyletic group (Figure 2). The Pacific Northwestern clade was not supported in MrBayes analysis (but was recovered in our other analyses, including our Bayesian molecular dating estimates and haplotype network [see below]). Collapsing that node leads to a series of monophyletic lineages that stem from a basal polytomy for the species. Hereafter, we refer to these collective lineages as the Pacific Northwestern clade as a matter of convenience. Two well supported clades are nested within *R. balteatus* (Figure 2): One clade corresponds to individuals sampled from the Bonneville Basin and Upper Snake River drainage (Figure 3), and is hereafter referred to as the Bonneville/Upper Snake Clade. The other, comprising individuals from inland populations along the Columbia Plateau and British Columbia (Figure 4), is hereafter referred to as the Northern Inland Clade. A sister relationship between the Northern Inland Clade and the Bonneville/Upper Snake Clade was supported by Bayesian analysis, but not by ML analysis (Figure 2). Collapsing that node would lead to a basal polytomy with Pacific Northwestern lineages, the Northern Inland Clade, and the Bonneville/Upper Snake Clade all stemming out of it. One individual sampled from the Bonneville system (BL 7300) carried a haplotype that is one base different from the most widespread haplotype found in the Northern Inland Clade (see Figures 1 and 6). Five other populations (Big Bear Creek, ID; Coeur d’Alene River, ID; Salmon River, ID; South Fork John Day River, OR; Yakima River, WA) contained individuals carrying divergent haplotypes from the Pacific Northwestern Clade and the Northern Inland Clade (see Figure 1).

A haplotype network shows intraspecific genetic variation within and among clades (Figure 6). Bonneville Basin/Upper Snake River haplotypes are separated from Pacific Northwestern haplotypes by 15 steps, and southern Bonneville haplotypes are separated from northern Bonneville/Upper Snake River haplotypes by 12 steps. Northern Bonneville/Upper Snake River haplotypes exhibit a star-burst pattern. Haplotypes from the Northern Inland Clade are separated from haplotypes in the Pacific Northwestern Clade by 16 steps. A number of Northern Inland Clade haplotypes differ by one or two base pairs from the most widespread shared haplotype, and also exhibit a star-burst pattern, whereas other haplotypes within this clade (DB9254 and DB9255) are as divergent from the Northern Inland haplotype as they are from the Pacific Northwestern haplotypes. Some of the haplotypes from the Pacific Northwestern lineages (SS9741 and SS9745) are 10 bp divergent from the other Pacific Northwestern haplotypes as well.

### Molecular dating estimation

**Figure 7:**
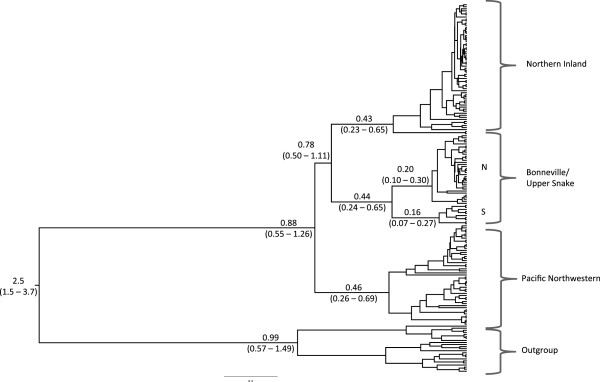
**Divergence time estimates.** Tree showing divergence time estimates for redside shiner clades. The Bonneville Clade contains two distinct clades, one corresponding to northern Bonneville/Upper Snake populations (N), and one corresponding to southern Bonneville populations (S). Mean divergence time estimates are listed above branches and 95% credible intervals are in parentheses below branches. All divergence time estimates are given in millions of years, and thus all intraspecific divergences are Pleistocene in age.

Molecular dating estimates show that divergence between *R. balteatus* and *R. egregius* occurred approximately 2.5 Ma (Figure 7), well within the 95% credible intervals for other divergence time estimates between the species [55,57]. Divergence within *R. balteatus* began approximately 0.88 Ma (Figure 7). Diversification within the Bonneville/Upper Snake, Pacific Northwestern, and Northern Inland clades began almost simultaneously, with divergence time estimates of 0.44 Ma, 0.46 Ma, and 0.43 Ma, respectively (Figure 7).

### Historical demography

**Figure 8:**
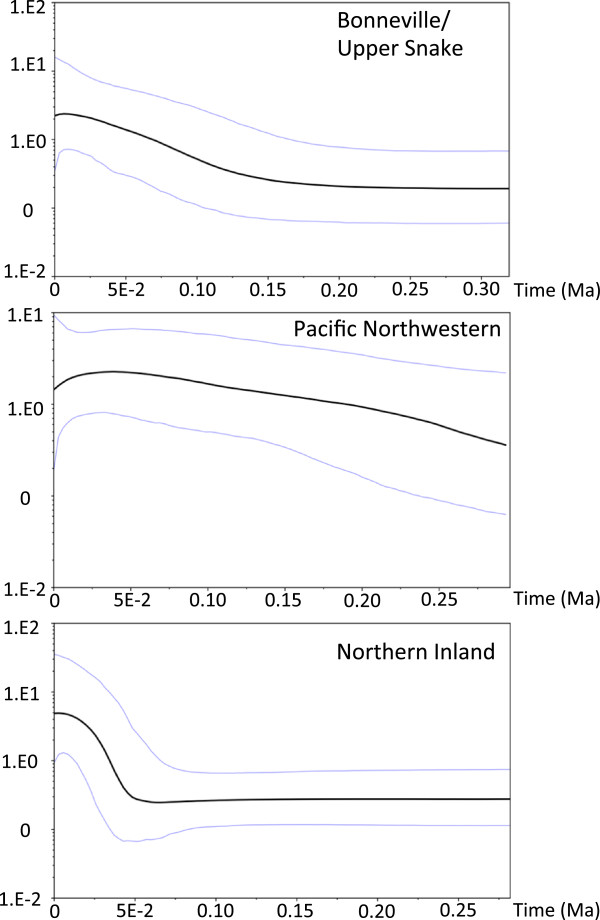
**Skyline plots.** Bayesian skyline plots showing the demographic history for each of the three clades of redside shiner. Black lines represent mean population sizes plotted over time, and blue lines represent 95% confidence intervals surrounding those means. All three clades show a signature of population expansion, albeit beginning at different times in the Pleistocene. The Northern Inland Clade shows the most recent (and most pronounced) expansion beginning ~50,000 years ago.

Bayesian skyline plots indicate that each of the three major clades experienced demographic expansions during the Pleistocene (Figure 8). The Bonneville/Upper Snake clade began expanding approximately 150,000 years before present, the Pacific Northwestern lineages were expanding prior to 300,000 years ago, and the most pronounced signature of expansion occurred in the Northern Inland clade, which began expanding approximately 50,000 years ago.

## Discussion

Genetic diversification within *R. balteatus* occurred well within the Pleistocene, however most of it does not appear to have been associated with the LGM. Our results are inconsistent with a late Pleistocene colonization of the Bonneville Basin during the Bonneville Flood. Rather, molecular dating estimates show that divergence between northern and southern Bonneville clades was likely much earlier, approximately 440,000 years ago, and that the northern and southern Bonneville clades began diversifying 200,000 and 160,000 years before present, respectively (Figure 7). Despite the uncertainty surrounding molecular dating using mtDNA, the 95% credible intervals surrounding these divergence time estimates did not include the Late Pleistocene when the Bonneville Flood occurred. It is plausible that *R. balteatus* entered the Bonneville Basin during an earlier flooding event that connected the Snake River to the Bonneville Basin [82]. The earliest margin of the 95% credible interval of this divergence time estimate overlaps with the presence of a large lake that formed in the Bonneville Basin approximately 650,000 years ago [83], although it remains unclear whether the catastrophic flooding event proposed by Ore [82] occurred at that time. The star-burst pattern of northern Bonneville and Upper Snake River haplotypes on the haplotype network (Figure 6) is suggestive that some northward dispersal from the Bonneville Basin into the Snake River at the time of the Bonneville Flood may have occurred. The development and use of more fine scale genetic markers would be useful in addressing this hypothesis.

Post-glacial colonization of most British Columbia sampling locations appears to have been from a single refugium in the Upper Columbia River drainage, with the lone exception being the Alouette Lake population east of Vancouver, BC. Individuals from Alouette Lake, which is in a Pacific Coastal drainage basin, were affiliated with lineages in the Pacific Northwestern clade (see Figure 1). The inland British Columbia populations all carried Northern Inland clade haplotypes (Figure 1), and the Northern Inland clade exhibited a pronounced signature of recent rapid demographic expansion (Figure 8). The star-burst pattern of many closely related haplotypes (Figure 6) is consistent with this scenario. The low genetic diversity in these populations in previously glaciated areas is consistent with the prediction that genetic diversity should be low after having undergone recent rapid expansion. Hence, the phylogeographic scenario proposed by McPhail and Lindsey [18], wherein *R. balteatus* colonized British Columbia from a refugium in the Upper Columbia drainage via a series of connections between glacial lakes, is plausible. The Clearwater and Salmon Rivers of northern Idaho have been identified as Pleistocene refugia [32,33,35,84], and could have sheltered *R. b. balteatus* as well.

Secondary contact between Northern Inland clade and Pacific Northwestern lineages appears to have occurred in five populations in the Columbia River drainage (Coeur D’Alene River, Clearwater River, Salmon River, South Fork John Day River, and Yakima River; see Figure 1). We postulate that this secondary contact is relatively recent because there is no apparent admixture of mtDNA haplotypes in the populations that we sampled in British Columbia, suggesting that post-glacial colonization occurred prior to the two clades coming back into contact with one another. Moreover, several populations that are isolated by physical barriers to gene flow do not appear to have mixed mtDNA haplotypes. For example, coastal drainages (i.e., Elk Creek, Hunter Creek, Satsop River, Siuslaw River and Umpqua River) have not been connected to the main drainage of the Columbia Basin since the end of the Pleistocene [47], so gene flow has not been possible in recent times. Other populations (i.e., Callapooia River, Dragoon Creek and North Fork Palouse River) occur above barrier falls, or above seemingly impassable rapids, such as Hurry Back Creek (above Hell’s Canyon along the Snake River). Such barriers seem to have prevented migrants from reaching these populations. Non-admixed populations above these barriers suggest that expansion of the clades did not occur until after the formation of these barriers. Palouse Falls (North Fork Palouse River), which formed during one of the more severe events associated with the Missoula floods, has a population above it containing only individuals affiliated with the Pacific Northwestern lineages. Another population in the Willamette Valley in Oregon (the Callapooia River, which is isolated from the lower Columbia River by another barrier falls) also contained haplotypes from the Pacific Northwestern lineages, so it is probable that Pacific Northwestern lineages were widespread during the Pleistocene. If the Missoula floods themselves were responsible for transferring individuals from the Northern Inland clade into habitats occupied by the Pacific Northwestern lineages, it does not appear to have done so during the most severe events such as the one that filled the Willamette Valley, otherwise the Callapooia River population above the falls would be expected to show genetic admixture as well, yet it does not.

It warrants mentioning that our sample sizes for each population are small (4 to 5 individuals per population), and may not have been sufficient to detect rare haplotypes in some areas. Additional sampling may reveal that non-admixed populations above barriers also experienced secondary contact that we did not detect with our limited sampling. Additional sampling may also reveal areas in the Fraser River drainage where the Northern Inland Clade and Pacific Northwestern lineages have come into secondary contact. Individuals from a lower Fraser River population (Alouette Lake) have Pacific Northwestern haplotypes, whereas individuals from upper Fraser River populations (Hay Creek, Nadsilnich Lake, and Shumway Lake) carry Northern Inland haplotypes. This sampling bias is unlikely to change our conclusion that the inland British Columbia populations dispersed from a refugium in the Upper Columbia River drainage because all individuals from those populations had haplotypes associated with the Northern Inland Clade.

If *R. b. balteatus* did survive the Pleistocene in multiple refugia, as our data suggest, the locations appear to be in the lower Columbia River system and/or along the Pacific Coast, and somewhere in the Upper Columbia River drainage, as noted above. This scenario fits the biogeography of several other taxa. The lower Columbia River itself has been identified as a refugial area for various taxa [29-31,36,85-87], as have areas along the Pacific Coast, including the Chehalis River Valley in Washington State [4,31,88]. Unique haplotypes (SS9741 and SS9745) occur in the Satsop River within the Chehalis River Valley (Figure 6), but others (SS9742, SS9743, and SS9744) were more closely affiliated with other populations along the Pacific Coast. The existence of the two divergent haplotypes in the Satsop River, as well as the three that are associated with other Pacific Northwestern lineages suggests that the Chehalis River Valley may have been invaded by *R. b. balteatus* individuals from other areas, representing a unique instance where rather than expansion out of a Pleistocene refugium, the refugium may have been invaded instead. The Pacific Northwestern lineages were likely widespread during the Pleistocene given that they showed the earliest signs of population expansion (Figure 8), and that they range from the Pacific coastal drainages to as far inland as the North Fork Palouse River in eastern Washington (i.e., the non-admixed population above Palouse Falls; Figure 1). Similarly, the Northern Inland Clade appears to have been widespread with populations ranging from the lower Snake River in southern Idaho to the upper Columbia drainage (Figure 1), although it appears that only the upper Columbia populations contributed to the post-glacial colonization of inland British Columbia.

The timing of the Northern Inland Clade’s expansion corresponds to the Wisconsin Glacial period (12 – 110 Ka) and into the Holocene, which is consistent with the idea of post-glacial expansion into inland British Columbia as the glaciers retreated. Population expansion of the Bonneville/Upper Snake Clade appears to be associated with the Illinoian Glacial period (130-200 Ka), or perhaps the Sangamonian interglacial (110-130 Ka), which is not consistent with a late-Pleistocene invasion at the time of the Bonneville Flood. Expansion of the Pacific Northwestern lineages appears to be associated Pre-Illinoisan glacial cycles (>200 Ka), as does the timing of the initial diversification within the species.

A single fish (BL7300) carrying a haplotype that nested well within the Northern Inland clade was sampled in a northern Bonneville Basin stream at the Utah/Idaho border. It may have been a “bait-bucket” transfer because no recent hydrological connections between that location (Blue Creek) and the middle Snake River are known and the required overland dispersal event from the nearest Columbia River location with its closely related haplotype (Salmon River) is over three hundred km. Such “bait-bucket” transfer incidents appear to be limited in our dataset, but are well known between the Bonneville Basin and Colorado River drainage of Utah [89,90]. Nevertheless, the biogeography of *Richardsonius* reflects historical distributions and drainage connections rather than recent anthropogenic introductions through most of its range, as is presumed to be the case for the majority of western North American freshwater fishes [60].

## Conclusions

Diversification among redside shiner clades occurred during the Pleistocene, but the early divergences do not appear to be associated with the LGM. It is unlikely that the species entered the Bonneville Basin at the time of the Bonneville Flood as postulated by Hubbs and Miller [54]. All but one of the British Columbia populations are related to Upper Columbia River populations with the exception of Alouette Lake east of Vancouver, which is more closely related to coastal populations of redside shiner. Hence, the biogeographic scenario outlined by McPhail and Lindsey [18] is plausible. These conclusions are based on mtDNA data, and analyses using additional unlinked markers would greatly enhance our understanding of this system.

## Availability of supporting data

The data set supporting the results of this article is available in the Dryad Digital Repository [doi:10.5061/dryad.k2c2p] [91].

## Competing interests

The authors declare that they have no financial or non-financial competing interests.

## Authors’ contributions

DDH, DKS, and BRR designed the study. DDH obtained field samples and generated DNA sequence data. DDH and BTS performed the data analyses. All authors contributed to the writing of the manuscript. All authors read and approved the final manuscript.
